# *Mycobacterium marinum* as a model for understanding principles of mycobacterial pathogenesis

**DOI:** 10.1128/jb.00047-25

**Published:** 2025-04-30

**Authors:** Aruna R. Menon, Rebecca J. Prest, David M. Tobin, Patricia A. Champion

**Affiliations:** 1 Department of Molecular Genetics and Microbiology, Duke University School of Medicine 12277 , Durham, North Carolina, USA; 2 Department of Biological Sciences, University of Notre Dame 6111, Notre Dame, Indiana, USA; 3 Department of Integrative Immunobiology, Duke University School of Medicine 12277 , Durham, North Carolina, USA; 4 Eck Institute for Global Health, University of Notre Dame 550321, Notre Dame, Indiana, USA; Geisel School of Medicine at Dartmouth, Hanover, New Hampshire, USA

**Keywords:** *Mycobacterium marinum*, pathogenesis, granuloma, ESX-1, model, TB, mycobacterium

## Abstract

*Mycobacterium marinum* is a fish pathogen that has become a powerful and well-established model that has accelerated our understanding of the mechanisms of mycobacterial disease. *M. marinum* is a versatile surrogate for understanding the closely related human pathogen *M. tuberculosis*, which causes tuberculosis in humans. *M. marinum* has defined key mechanisms of pathogenesis, both shared with *M. tuberculosis* and unique to this species. In this review, we discuss the discovery of *M. marinum* as an occasional human pathogen, the shared aspects of pathogenesis with *M. tuberculosis,* and how *M. marinum* has been exploited as a model to define the molecular mechanisms of mycobacterial pathogenesis across several phases of infection.

## INTRODUCTION AND HISTORICAL PERSPECTIVE

Tuberculosis (TB) has burdened human health for thousands of years (1, 2). However, the causative bacterial agent, *Mycobacterium tuberculosis,* was discovered in 1882 (3). Within 40 years of the discovery of *M. tuberculosis,* the Bacillus Calmette-Guérin (BCG) vaccine was developed and administered (4). Concurrent with these discoveries, they are exploited as a model to define the molecular mechanisms of mycobacterial pathogenesis across several phases of infection.

Regarding human diseases, there were several reports of tuberculosis-like disease in cold-blooded animals, including fish (5–8), turtles (9), and snakes (10). The isolation of the etiologic agents of the observed diseases led to new non-tubercular mycobacterial species including *M. marinum,* which was also identified as *M. balnei* (7, 11). *M. marinum* was isolated in 1926 from saltwater fish after an outbreak of “spontaneous tuberculosis” at the Philadelphia Aquarium (7). Soon after, *M. balnei* was isolated from skin lesions in humans from swimming pools, popularizing the terms “swimming pool granuloma” and “sore elbows” (11–13). Later case studies described infections involving injuries obtained while tending fish tanks (12). Further case studies and strain isolations revealed that *M. marinum* and *M. balnei* were the same species, and the *M. balnei* strain was subsequently subsumed by *M. marinum* (14).

By the 1960s, Clark and Shepard observed the effects of *M. marinum* infection on a large variety of poikilothermic species and mice. They concluded that *M. marinum* grew optimally between 25 °C and 33 °C, was easily studied in poikilotherms, and could cause systemic infection following intravenous inoculation of mice (14–16). Importantly, as early as the 1950s, it was noted that patients with swimming pool granulomas often tested positive for a tuberculin patch test, suggesting a clear commonality between *M. marinum* and *M. tuberculosis* infection and providing the first hints that *M. marinum* could be used as a model for *M. tuberculosis* infection (13, 17).

These early studies showed that *M. marinum* has several attributes that make it easier to manipulate in the laboratory (Table 1). For these reasons, a 1994 study was one of the first to use *M. marinum* as a direct model for *M. tuberculosis* pathogenesis (15). This hallmark paper by Ramakrishnan and Falkow showed that *M. marinum* was associated with mouse macrophages and epithelial cells and persisted with cytopathic effects, suggesting that *M. marinum* mirrors central aspects of the infection cycle of *M. tuberculosis* in the human lung.

**TABLE 1:** Characteristics of *M. marinum* and *M. tuberculosis*

	*M. marinum*	*M. tuberculosis*	Reference(s)
Growth temperature	25-33°C	37°C	(14)
Doubling time	~4–8 h	~20 h	(7, 14)
Transmission	Water-borne	Airborne	(18, 19)
Biosafety level	2	3	(15, 20)
Genome size	6.5 Mbp	4.4 Mbp	(21)
Replicates within specific host cells	Yes	Yes	(15)
Common clinical presentations	Localized, cutaneous, painless lesions on hands or feet	Acute: malaise, fever, weight loss, lesions present in the lungs	(19, 22–24)
Diagnostic tools	Clinical history (exposure to aquatic environments, etc.) Culture from a tissue biopsy Histopathology PCR for *M. marinum* PPD skin test Acid-fast stain on tissue samples	Xpert MTB/RIF Assay Sputum microscopy analysis Culture Blood test (Interferon-gamma release assay) PPD skin test Chest XRAY	(19, 22–24)

As the field approached the late 20^th^ century, molecular techniques, including PCR and direct genome sequencing, revealed that *M. marinum* is one of the most closely related species to the larger *M. tuberculosis* complex (MTBC), a group of closely related species that cause TB (25). Several papers using *M. marinum* revealed the fundamental aspects of physiology and pathogenesis in the early 2000s (21, 26–34). Indeed, according to PubMed, yearly citations including *“M. marinum”* increased from 28 references in 2000, to ~40 references in 2010, to 93 references in 2024, reflecting increased adoption of this organism as a model system. A key paper by Stinear et al. moved *M. marinum* studies to the mainstream of the mycobacterial field, suggesting that *M. marinum* and members of the MTBC species diverged from a common ancestor, highlighting shared cell envelope lipids, virulence factors, and secondary metabolism genes between the two species (21). Following this paper, the field saw the first iterations of a searchable database of the *M. marinum* genome, including gene ontology and protein prediction, known as Marinolist, which followed Tuberculist (35), the forerunner to Mycobrowser (36).

**Fig 1:**
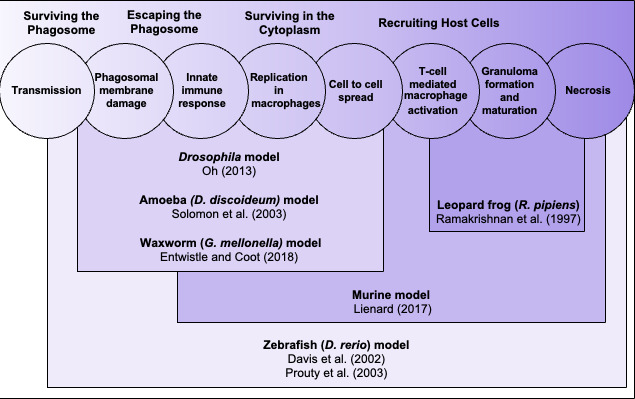
*M. marinum* contributions to understanding the principles of mycobacterial pathogenesis. *M. marinum* studies have contributed to key aspects of mycobacterial pathogenesis (circles) across both early and chronic stages of infection. Seminal papers introducing key host models are represented, highlighting their contributions.

In addition to bacterial genetics, animal models have been extremely powerful in understanding mycobacterial infection. Figure 1 highlights the key stages of *M. marinum* infection and the host models that have contributed to understanding pathogenesis. Briefly, during early infection, the mycobacteria must survive and replicate in phagocytic host cells through phagosomal maturation arrest and membrane damage, and modulation of the innate immune response. During chronic infection, there is recruitment of host cells for mycobacterial cell-to-cell spread, macrophage activation, and ultimately granuloma formation and maturation. Because *M. tuberculosis* is an obligate human pathogen, many aspects of TB infection need to be studied *in vivo* and require animal models. However, although several animal models for *M. tuberculosis* infection exist to study disease (mice, guinea pigs, rabbits, and non-human primates), these animals are not natural hosts of *M. tuberculosis* (37–39). Early studies using *M. marinum* leveraged its natural hosts, including the leopard frog (*Rana pipiens*) and the zebrafish (*Danio rerio*), in which the bacteria establishes and maintains chronic infection, making them ideal models for long-term TB disease (40). Indeed, unlike infection of mice and chick embryos with *M. marinum,* which resulted in disseminated and acute disease, infection of frogs or fish resulted in the formation of granulomas, one of the most notable features of *M. tuberculosis* infection (16, 41, 42). Granulomas are complex immune structures that can wall off or isolate the bacteria. They form early during *M. tuberculosis* infection, when macrophages cluster and undergo cell death, releasing cytokines to attract other innate immune cells (43, 44). Although granulomas are extremely dynamic, the canonical TB granuloma consists of a necrotic, mycobacteria-filled center, surrounded by macrophages (epithelioid, foamy, and inflammatory) and neutrophils and further bordered by adaptive immune cells and fibroblasts (45–47). Although granulomas serve as a host-pathogen interface that can restrict infection, they can also allow bacterial replication, dissemination, and transmission, as well as reduce susceptibility to treatment (47–49). Both superinfecting *M. marinum* and *M. tuberculosis* localize to already established granulomas by the trafficking of infected macrophages (50, 51).

Other model organisms used with *M. marinum* include *D. melanogaster* (52)*, Dictyostelium discoideum* (53)*,* and *Galleria mellonella* larvae (54, 55). It is unclear how well these models recapitulate later aspects of the mycobacterial cell cycle as they lack an adaptive immune response (52, 56–58). As such, infection of these organisms with *M. marinum* is generally used to model early bacterial growth within phagocytes (58, 59). However, the zebrafish (*Danio rerio*) model of infection effectively models the entire mycobacterial cycle of *M. tuberculosis* in the human lung (60). A 2002 study was the first to leverage the zebrafish as a natural host for *M. marinum* infection (61). It should be noted that like other animal models, there are some limitations to using zebrafish. Zebrafish lack lungs and thus cannot exactly replicate the process of *M. tuberculosis* infection in human airways. In addition, there are limited antibodies for zebrafish, limiting molecular biology experiments such as flow cytometry and immunohistochemistry (62–64). However, due to the optical transparency and genetic manipulability of zebrafish, there are many fluorescent transgenic lines that allow not only quantification but also visualization of the mycobacterial infection process (65). Numerous studies have demonstrated that zebrafish larvae infection with *M. marinum* parallels early *M. tuberculosis* infection by creating an actively replicating niche within macrophages (40, 60, 65–67). Similarly, in adult zebrafish, *M. marinum* infection results in caseating and necrotic granulomas that are structurally comparable with those formed in tubercular human lungs (40, 65, 68, 69). In the leopard frog, *Rana pipiens,* although granulomas do form, the lesions are not necrotic or caseating. Additionally, *R. pipiens* is an outbred species with high genetic diversity between individual animals, which likely causes higher variability within experiments (16, 40). For these reasons, the scientific community has adopted the zebrafish-*M. marinum* infection model for studying both early and chronic stages of *M. tuberculosis*.

These early studies showed that the virulence and physiology of *M. marinum* are closely related to *M. tuberculosis* at the molecular level (25, 32–34). In addition, pathogenesis could be easily modeled in macrophages or in zebrafish in a BSL2 laboratory, without the need for a BSL3 facility. The Mycobrowser (Marinolist) database and the use of proteomics combined with burgeoning molecular and genetic tools, including transposon screening, primed *M. marinum* as a model system for understanding *M. tuberculosis* pathogenesis and mycobacterial physiology at large.

## MOLECULAR DETERMINANTS OF MYCOBACTERIAL INFECTION

Once inside the host, intracellular mycobacterial survival is a key aspect of establishing infection (33, 70–72). Molecular biology studies that use genetic deletions, proteomics, and transcriptomics have greatly increased the field’s understanding of basic mycobacterial physiology and genetics. In addition, important aspects of the host response to mycobacterial infection have been discerned and complemented work done in *M. tuberculosis* using *M. marinum* in cell culture and animal models (37, 60). Examples of our understanding of key virulence determinants and host responses that were positively impacted by using *M. marinum* as a model are outlined below.

### Surviving the phagosome

Once inside the macrophage, mycobacteria initially reside in the phagosome. Within this niche, mycobacteria combat cellular stresses and phagosome-lysosome fusion, which results in acidified compartments that degrade pathogens (33). In addition to acid stress, toxic levels of copper and zinc cations are shuttled into the phagosomal compartment as part of the host response to mycobacterial infection (73–78). To combat this, copper transporters and efflux pumps make up a complex homeostasis system, including mycobacterial copper transport protein B (MctB), copper-transporting P1-type ATPases (CopA, CtpA, CtpB, and CtpV), a copper metallochaperone (CopZ), a copper sensing repressor (CsoR), and mycobacterial metallothionein (MymT) (73–75, 79). A recent study used *M. marinum* to characterize a newly discovered copper uptake transporter, MMAR_0267 (Rv0102), finding links between copper metabolism and host cell apoptosis (75). Similarly, zinc is exported from the mycobacterial cell by CtpC, a Zn^2+^ efflux pump with isoforms present in both *M. tuberculosis* and *M. marinum* (77, 78). In the *M. marinum-D. discoideum* model, CtpC is upregulated upon increased Zn^2+^ in the mycobacterium-containing vacuole to prevent zinc poisoning (78).

It was initially thought that pathogenic mycobacteria such as *M. tuberculosis* and *M. marinum* survived in the phagosome exclusively through blocking phagolysosome fusion. However, both *M. marinum* and *M. tuberculosis* reside within the acidified phagosome and resist the microbicidal effects (71, 80–82). Infections of *M. marinum* in zebrafish showed that blocking phagolysosome fusion is only partially successful in aiding bacterial survival (83). Indeed, acid resistance mechanisms of mycobacteria have been extensively documented (80, 84). For example, the *marP* gene has been implicated in *M. tuberculosis* acid resistance, as *M. tuberculosis marP* mutants are attenuated in mice and are unable to grow in acidic environments (85). In these studies, it was unclear whether these phenotypes were due to impaired acid resistance or susceptibility to other antimicrobial effectors encountered *in vivo* (83, 85). However, the zebrafish-*M. marinum* model showed that a *marP* mutant was only increasingly cleared when the bacteria were transported to the phagolysosome, showing that *marP* is directly implicated in maintaining intrabacterial pH and serves as an important bacterial mediator in surviving this aspect of infection (83).

### Escaping the phagosome: ESX-1 and PDIM

The Bacille Calmette-Guérin (BCG) vaccine remains the only widely used TB vaccine (18, 86). However, it is primarily effective only for childhood TB disease, does not provide strong protection from primary infection in adults, and cannot be administered to immunosuppressed individuals (18). BCG is a live-attenuated strain of *Mycobacterium bovis,* a virulent mycobacterial species that is a member of the MTBC. There are three BCG-specific regions of difference (RDs) between the BCG vaccine, and *M. bovis* and *M. tuberculosis* (87, 88). The RD1 region, which encodes for the ESX-1 (ESAT-6-system-1) secretion system, is considered the major attenuating genomic deletion in the BCG vaccine strain (89). ESX-1 is a conserved type VII secretion system, which transports antigenic virulence factors onto the mycobacterial cell surface and into the host (27, 90, 91). ESX-1 secretion is required for phagosomal escape. Strains lacking ESX-1 are retained in the phagosome and attenuated (92).

The ESX-1 secretion system is highly conserved between *M. tuberculosis* and *M. marinum* (93). Accordingly, *M. marinum* has been used extensively as a model organism for studying ESX-1 regulation and secretion, as reviewed by Chirakos et al. (93). The ESX-1 secretion system is intricately regulated, and its function and regulation have been linked to broad cellular pathways including metabolism, motility, and biofilm formation (94–96). WhiB6 was originally discovered as a positive regulator of ESX-1 gene transcription in *M. tuberculosis* clinical isolates (97). However, much of the subsequent characterization of WhiB6 was performed using *M. marinum* through molecular genetics, transcriptomics, and the zebrafish infection model (96, 98, 99). Other regulatory elements of ESX-1 have been discovered and studied in *M. marinum* (26, 94, 100–104).

Six conserved components (EccA-E, Esx-conserved component) are required for transporting protein substrates from the mycobacterial cytoplasm across the cytoplasmic membrane. Five of the six components of the membrane complex are encoded across all the ESX systems (105). However, it is unknown how the secreted substrates of each distinct ESX system are transported through the periplasm and outer mycolate outer membrane (MOM). We recently used *M. marinum* strains with targeted genetic deletions of each known ESX-1 substrate gene and performed proteomic analysis on the secreted protein fractions (106). By comparing which ESX-1 substrates were secreted in each deletion background, we constructed a proposed order of ESX-1 substrate secretion (106). Not only does this work provide a blueprint for how the substrates may span the mycobacterial cell wall (106) but it also provides a clearer picture of why EsxA (ESAT-6) is essential for ESX-1 function. It was originally thought that EsxA was the ESX-1 lysin, but later work suggested that although EsxA is required for ESX-1 substrate secretion, it is other substrates that promote phagosomal lysis (100, 107). Our study complemented this work, showing that EsxA is part of a group of early secreted substrates that are required for the secretion of three distinct groups of later secreted substrates (106). The use of *M. marinum* in this work was crucial, as the generation of numerous unmarked deletion and complementation strains with cell-associated and secreted protein fractions would be time-consuming with *M. tuberculosis*. Several studies have followed this paper using both *M. marinum* and *M. tuberculosis*, demonstrating direct interactions between ESX-1 substrates that support the proposed hierarchy (103, 108). Moreover, a recent study in *M. tuberculosis* localized EsxB (CFP-10, Culture Filtrate Protein, 10 kDa), an early secreted substrate required for the secretion of later substrates, to the mycobacterial periplasm (109).

Additional studies have used *M. marinum* to probe the modification of ESX-1 substrates. The ESX-1 secreted substrate EsxA (ESAT-6, Early Secreted Antigen, 6 kDa) was one of the first identified N-terminally acetylated bacterial proteins in 2004 (110). EsxA is N-terminally acetylated in both *M. marinum* and *M. tuberculosis,* and studies in both species have linked the acetylation status of EsxA to virulence (111, 112). However, we recently published a study using *M. marinum,* which finally identified the conserved Emp1 N-acetyltransferase (NAT) responsible for the N-terminal acetylation of EsxA and other proteins (111, 113). Interestingly, the *M. marinum* strain lacking the Emp-1 NAT secreted ESX-1 substrates during growth *in vitro*. However, during macrophage infection, the strain was delayed in escaping the phagosome, resulting in a loss of macrophage cytolysis and cell-to-cell spread. Overall, however, the role of N-terminal acetylation in mycobacteria remains an emerging field, as few other mycobacterial NATs have been characterized. One exception is Arylamine N-acetyltransferase, an anti-TB drug target. The *M. marinum* arylamine NAT was used as a model for studying the enzyme’s active site and screening anti-TB compounds (114–116).

In addition to ESX-1, the unique lipids in the mycobacterial cell envelope have key roles in virulence and survival during infection. Phthiocerol dimycocerosate (PDIM) and phenolic glycolipids (PGLs) are unique mycobacterial outer lipids that are conserved between *M. tuberculosis* and *M. marinum*. PDIM has a variety of roles during mycobacterial infection, including cell envelope impermeability, phagosomal escape, and overall immune response (117–120). PDIM is important during early infection by both *M. tuberculosis* and *M. marinum,* with PDIM-deficient strains often reported to have decreased or abrogated virulence (120–124). Indeed, it has been suggested that PDIM enhances ESX-1-dependent phagosomal permeabilization and access to the macrophage cytosol in several studies of *M. tuberculosis* (120, 125). Using PDIM-deficient strains of *M. marinum,* we demonstrated that protein secretion was broadly decreased, with the largest effects on the EspE and EspF ESX-1 substrates (117). By further exploring the assembly and interaction of ESX-1 with and within the mycobacterial cell wall, the field is moving closer to understanding the precise molecular mechanism of phagosomal lysis.

PDIMs and PGLs have also been implicated in mycobacterial biofilms. Biofilm formation by pathogenic bacteria often leads to extended, multi-drug treatment and contributes to drug tolerance (126). Both *M. tuberculosis* and *M. marinum* exhibit a characteristic biofilm-like cording morphology during growth on agar and clumping and adhesion to surfaces in liquid culture (127–129). Cording has also been observed *in vivo*, specifically shown in immunodeficient zebrafish where bacteria grow extracellularly (44, 130). Further work characterized the formation, physiology, and components, investigating if cording is representative of mycobacterial biofilm formation, as reviewed previously (131). *M. marinum* strains lacking PDIMs and PGLs have altered cell surface properties, which may cause decreased biofilm formation (132). Biofilm-deficient mutants generally have decreased persistence in macrophage and murine infections (128, 129, 133). Biofilms offer protection from the host immune system, increased persistence in infection, as well as tolerance to antibiotics (131, 132, 134, 135). *M. marinum* has been used as a model for the identification of the surface-exposed proteins of mycobacterial biofilms. Proteomic studies identified how cell- and biofilm-surface proteins change over time and biofilm subtype (135, 136). One study proposed using synthetic nanobodies against identified surface-exposed proteins to deliver targeted drug treatments, thereby increasing the effectiveness of TB treatments (136).

There may also be a link between PDIM, PGL, and iron uptake. Both *M. marinum* and *M. tuberculosis* produce two specialized iron-scavenging siderophores, carboxymycobactin and mycobactin, which are secreted by the ESX-3 secretion system into the extracellular environment or remain attached to the mycobacterial cell wall, respectively. *M. tuberculosis* and *M. marinum* strains that cannot produce or secrete mycobactins display reduced virulence (137–142). In *M. marinum,* the mycobactin synthase K (MbtK) lysine acetyltransferase is required for mycobactin production, as well as PDIM and PGL synthesis (117, 138). The deletion of *mbtK* reduced virulence, which can be restored by overexpression of PapA5, a PDIM/PGL biosynthesis protein, or Eis, an N-acetyltransferase that modifies aminoglycoside antibiotics and mycobacterial proteins (117).

Together with *M. tuberculosis,* the use of *M. marinum* has contributed fundamental knowledge regarding how the ESX-1 system and surface-associated mycobacterial lipids promote protein secretion and phagosomal lysis.

### Surviving in the cytoplasm: protein secretion and manipulating the immune response

In addition to the ESX-1 system, pathogenic mycobacteria share four other Type VII ESX secretion systems (ESX-2–5), as described thoroughly in several reviews (105, 143–146). The ESX-3 and ESX-5 secretion systems are required for bacterial growth *in vitro* under certain conditions because of their roles in nutrient transport (119, 147). Studies in both *M. marinum* and *M. tuberculosis* showed that the ESX-5 secretion system is also required for virulence and maintenance of the mycobacterial cell wall (147). Work in *M. tuberculosis* revealed that the ESX-2 and ESX-4 systems do not have clearly defined roles, but these systems may work with ESX-1 to promote phagosomal membrane permeabilization and with ESX-5 to promote secretion of the CpnT toxin (148, 149). In addition to CpnT, the ESX-5 system secretes many protein substrates, including PE/PPE and PE_PGRS proteins (28, 150–152) and EsxM (153). PE/PPE and PE_PGRS proteins are specific to mycobacteria and were recently reviewed here (154, 155).

Where the ESX-1 system is crucial for mycobacterial survival in early infection, ESX-5 secretion establishes a moderate, persistent infection. ESX-5 works downstream of phagosomal lysis and influences inflammasome activation, IL-1β activation, and caspase-independent cell death (156, 157). ESX-5-deficient *M. marinum* strains are hypervirulent when tested in adult zebrafish, with increased induction of proinflammatory cytokines, such as TNF and IL-6 (156, 158). LipY, a PE (*M. tuberculosis*) or PPE (*M. marinum*) domain-containing lipase secreted by ESX-5, is involved in triacylglycerol (TAG) accumulation and host immune response (150, 151, 159). In addition, mycobacteria may exploit host lipids during intracellular growth through the secretion of Lip Y and other lipolytic proteins to break down host phospholipids into free fatty acids (160, 161). The inhibition of LipY during *M. marinum* infection in zebrafish decreased bacterial burden and is a proposed target for anti-TB drugs (151).

*M. marinum* has also been used to illuminate the impacts of strain-level differences in *M. tuberculosis,* which explain human-specific adaptations of TB-causing species. A recent example of this is the ESX-5-secreted early effector protein, EsxM. Modern lineage strains of *M. tuberculosis* have a truncated version of the EsxM protein, whereas the ancestral lineage strains and other pathogenic species of mycobacteria have a full-length version. Using *M. marinum*, we found that full-length EsxM protein promotes dissemination in macrophages and zebrafish and that humans infected with *M. tuberculosis* who had a full-length version of this protein were more likely to have higher rates of extra-pulmonary TB (153).

Although both *M. tuberculosis* and *M. marinum* access the macrophage cytosol following phagosomal rupture by the ESX-1 system (162), only *M. marinum* has been reported to engage in actin-based motility in some cell types (163–165). MirA, a PE_PGRS protein secreted by ESX-5, stimulates actin polymerization by interaction with host protein N-WASP (164). However, during infection of certain strains of *Dictyostelium discoideum,* both *M. marinum* and *M. tuberculosis* use host F-actin to create a barrel-shaped structure or “ejectosome,” at the plasma membrane, inducing non-lytic ejection from the host cell and allowing cell-to-cell spread (53, 166–168). These findings have prompted the further study of mycobacteria using host actin to proliferate and disseminate during infection (169–171).

Phagosomal permeabilization results in mycobacterial DNA and RNA in the cytosol, inducing the production of host type-I interferons (IFNs) during *M. tuberculosis* and *M. marinum* infection (91, 172). In addition, *M. marinum-* and *M. tuberculosis-*induced macrophage cytotoxicity is related to the release of lysosomal proteases such as cathepsin B during ESX-1-mediated phagosomal rupture (157). The expression of *M. marinum* ESX-1 in recombinant BCG induced a type-I IFN response in a proposed vaccine design (86). The *M. marinum* model has been used to further study the nuances of type-I IFN response to mycobacterial infection, including serine protease inhibitors (173) and IFNγ-mediated autophagy (174, 175). In addition, there is ESX-1-dependent expression of host microRNAs (miRNAs), which control the expression of immune response protein-encoding genes and display differential expression patterns during mycobacterial infection (176–181). These studies support that the mycobacterial ESX-1 secretion system promotes mycobacterial survival during early infection by manipulating the host immune response through the upregulation of pro-inflammatory cytokines.

### Interactions with host cells can combat, sequester, or spread mycobacterial infections

Several components of the ESX-1 secretion have been implicated in the formation of the granuloma during infection. As mentioned earlier, granuloma formation includes several steps. The early stages of the granuloma consist of macrophage clustering, forming aggregates of macrophages and other innate immune cells (43, 44). These aggregates turn into mature granulomas as macrophages undergo an epithelioid transformation, containing the bacteria within while recruiting neutrophils, T cells, and B cells and increasing the process of host angiogenesis (182, 183). During early infection, the ESX-1 system is particularly important for the early granuloma stages that allow for later progression into the mature structure (184, 185). *M. marinum* lacking the RD1 locus exhibit weakly formed, smaller aggregates of macrophages, indicating the importance of early mycobacterial effectors in the formation of the granuloma (186). Indeed, ESX-1-deficient *M. marinum* strains have reduced macrophage recruitment and aggregation of macrophages in zebrafish larvae at 5 days post-infection (43). To find other genes important for granuloma formation, a *M. marinum* transposon-insertion library was screened *in vivo* in zebrafish larvae, revealing two additional ESX-1 associated genes, *MMAR_5425 (espK*) and *MMAR_5456 (espL*). EspK is a secreted substrate of the ESX-1 system in *M. marinum,* which directly interacts with the EspB substrate, and is required for the secretion of the EspE and EspF substrates (31, 106, 184, 187). EspL is a conserved cytoplasmic ESX-1 protein. *M. marinum* strains with Tn-insertions in either *espK* or *espL* had lower survival in cell culture models compared with the WT strain and were defective in cell-to-cell spread in macrophages (26, 184). Additionally, EspL was required for wild-type EsxA (ESAT-6) secretion levels and EspE stability, which may likewise be important in early granuloma formation (26, 184, 186, 188). However, the role of EspK in mycobacterial granulomas is unclear, as EspK appears to be dispensable for virulence in *M. bovis* and *M. tuberculosis* (189–191). Further work in *M. marinum* will enable the investigation of the roles of specific ESX-1 proteins in granuloma formation.

The integrity of the mycobacterial cell envelope is important in granuloma formation. A recent study showed that an *M. marinum* strain with transposon insertion in *fadE33 (ipdE2*) was defective in granuloma formation in zebrafish (184). Although *fadE33* is a part of the cholesterol catabolism pathway, the loss of FadE33 did not alter the cell envelope lipid profile (184, 192). Pathogenic mycobacteria, including *M. marinum,* have a second, non-essential copy of the general Sec secretion system (193). *M. marinum* lacking the accessory SecA2 ATPase has structural differences in the cell envelope and forms fewer granulomas in zebrafish, a phenotype that is recapitulated in *M. tuberculosis in vivo* infections (194).

In addition to bacterial factors, the *M. marinum* zebrafish model has provided insight into host elements that are important during granuloma formation. Neutrophils are an important niche for mycobacterial infections and are one of the most abundant cell types in patients with active TB infections. Similar to macrophages, early recruitment of and bacterial interactions with neutrophils are important in the granuloma infection and infection. In the zebrafish model, neutrophils are protective at early stages of infection because they kill *M. marinum* through the production of oxidative compounds such as reactive oxygen species (ROS) and nitric oxide (NO) (195). These findings complement human observational studies where patients with reduced levels of NADPH-dependent oxidative bursts were more susceptible to mycobacterial infection (196). Additionally, oxidative compounds resulted in hypoxia, maintaining the neutrophil response and stabilizing hypoxia-inducible factors (HIF). The stabilization and upregulation of HIF-1α, or the downregulation of HIF-2α, decreased *M. marinum* bacterial burden in zebrafish. Indeed, HIF-1α stabilization resulted in increased inducible NO synthase (iNOS) expression and subsequent nitric oxide levels, whereas HIF-2α stabilization decreased levels, impacting neutrophil activation and pathogen clearance (195, 197, 198). These studies using *M. marinum* corroborated previous *in vitro* work on the regulation of these HIF isoforms to combat bacterial pathogens, as well as the role of activated neutrophils in mycobacterial infections (199–201).

Beyond the granuloma, the *M. marinum-*zebrafish infection model has revealed insight into the bacterial response to host immune cells. For example, the cell wall lipid PDIM masks the immune recognition and TLR detection of *M. marinum* invasion by specific macrophage populations (202). In *M. marinum* strains lacking PDIM, there was increased recruitment of activated macrophages rich in iNOS expression to the site of infection in the zebrafish infection model. Likewise, during *M. tuberculosis* infection, iNOS-mediated microbicidal activity was increased when PDIM-deficient *M. marinum* was infected into macrophages. Aerosol infection of mice with PDIM-deficient *M. tuberculosis* strains likewise resulted in the recruitment of greater numbers of iNOS-positive monocytes (202). In another study, PDIM-deficient *M. tuberculosis* was attenuated both in WT and iNOS-deficient mice (122). The importance of PDIM in mycobacterial survival to the host immune response has made it a potential vaccine target (203). At the same time, a related mycobacterial lipid, phenolic glycolipid (PGL), promotes the recruitment of permissive macrophages that allow for spread to different host tissues by exploiting chemokine signals acting through the chemokine receptor CCR2. Infection with *M. marinum* strains lacking PGL resulted in fewer macrophages being recruited to the site of infection, which was rescued by adding in the CCR2 ligand, CCL2. New methods have recently been developed to visualize PGL in live mycobacteria (204).

TNF, a potent pro-inflammatory cytokine, is an important host factor during mycobacterial infection (205). Interestingly, anti-TNF therapeutics for rheumatoid arthritis resulted in active TB infections in humans (206). Originally, TNF was considered important for granuloma formation because TNF-deficient mice had disorganized lesions (207–211). However, *M. marinum* infection of zebrafish larvae showed that TNF’s major impact on granulomas is through decreased microbicidal activity within macrophages and an acceleration of the overall infection timeline. Indeed, TNF-deficient animals still form early granulomas, but these are unstable and prone to necrosis (44). TNF-deficient animals also showed continued induction of other factors important in granulomas, such as host matrix metalloproteinase 9 (MMP9), which is highly expressed in the epithelioid population of mycobacterial granulomas and is necessary for their formation and maturation. This suggests that the initial phases of granuloma formation may be driven by TNF independent mechanisms, and indeed, fully deficient TNF patients are still able to form granulomas (188, 212, 213). Further work in mature granulomas formed during *M. marinum* infection of adult zebrafish showed that Type 2 inflammatory pathways, primarily driven by *stat6,* are required for macrophage epithelioid transformation and granuloma formation (214), and these Type 2 immune signatures are recapitulated in human and macaque models of granulomas (214–218).

*M. marinum* infection models have also revealed that host angiogenic signaling, a known feature of human granulomas, is important to bacterial burden and dissemination (219). Anti-angiogenesis drugs can also be combined effectively with front-line antibiotics (220). The *M. marinum* granuloma model has revealed both bacterial and host components that are important for this pathway. One of the more well-known components of the mycobacterial cell envelope, trehalose dimycolate (TDM), has been shown to be important for mycobacterial survival *in vivo*, including phagolysosome fusion, activation of metalloproteases, and modulation of the host immune response (221–223). However, studies using the *M. marinum* model showed that modifications to TDM by the enzyme proximal cyclopropane synthase of alpha-mycolates (PcaA) induce host angiogenesis in the early granulomas, allowing for bacterial growth and spread *in vivo* (219, 224). These signals were shown to be driven through macrophage activation of the NFAT pathway (219). Additionally, published single-cell data sets on *M. marinum*-infected granulomas provide data on multiple host pathways that might be targeted for new therapeutics (136, 185, 214). Further work to characterize the role of different host cells, including neutrophils, and adaptive immune cells, such as B and T cells, in granulomas using the *M. marinum-*zebrafish model promises to provide data on new anti-mycobacterial treatments.

## WHERE CAN *M. MARINUM* CONTRIBUTE NEXT?

There are many avenues of investigation remaining regarding the mechanisms underlying mycobacterial infections. The conserved and unique regulatory mechanisms, at the transcriptional and translational levels, that control mycobacterial infection remain a growing field of study (225). For example, leaderless mRNA translation, which is rare in *E. coli*, is common in *M. tuberculosis* and may impact pathogenesis (226, 227). In addition, some secreted proteins alter the host epigenome through post-translational modifications, as reviewed by Singh and Nagaraja (228). Additionally, TB granulomas are extremely diverse and dynamic structures, and there is evidence that the microenvironment of the granuloma can contribute to disease outcomes (49, 229). However, little is known about the contribution of the individual immune cells, like macrophages and neutrophils, to these different states. Recent work has shown the importance of Type 2 immune signaling for necrotic, organized granulomas using *M. marinum* with zebrafish transgenic lines and new *ex vivo* models (214). The power of fluorescently labeled immune cell lines in zebrafish with the growing number of large-scale data sets like single-cell RNA-seq will continue to allow the field to leverage *M. marinum* to illuminate *M. tuberculosis* infection processes.

Looking beyond canonical infection, one understudied aspect of TB disease is tuberculosis meningitis (TBM), which is a deadly extrapulmonary form of TB. Although only 1%–2% of TB cases progress to TBM, TBM infections have a 50% mortality and morbidity rate despite treatment (230–232). Because TBM is difficult to diagnose and treat, understanding the development of TBM could improve its outcomes. *M. marinum* can infect microglia and brain tissue in both cell culture and animal models, and therefore, it can be exploited as a model for TBM infections (233, 234). A study using the *M. marinum*-zebrafish infection model showed that mycobacteria can cross the blood-brain barrier, either as free bacteria or by trafficking of infected macrophages (235). This is supported by an earlier study that showed *M. tuberculosis* could traverse an *in vitro* blood-brain barrier by manipulation of the host-cell actin cytoskeleton (236). In addition, damage to brain endothelial cells by vasculitis is ESX-1-dependent (235). Initial experiments have shown that *M. marinum* infection of microglia resulted in increased secretion of pro-inflammatory cytokines, consistent with immunological profiles seen in humans with TBM (234). TBM remains an emerging field, with major gaps in the roles played by individual immune cells during infection, and the host inflammatory response as a whole, as well as limited treatment options for patients (237, 238).

Another field in which *M. marinum* is well-poised to contribute to is drug discovery. Rifampicin-resistant (RR-TB), multidrug-resistant (MDR-TB), and extensively drug-resistant tuberculosis (XDR-TB) cases are increasingly common, with an estimated 410,000 cases in 2023, making up 14.6% of all antimicrobial-resistant infections worldwide (18). Resistance to new drugs occurs quickly, and drug-resistant TB cases are difficult to diagnose and treat (18, 239). Therefore, there is a heightened demand for new treatments for TB infection. *M. marinum* has been used to screen drug libraries, allowing high-throughput screening with liquid-handling robots and instrumentation usually not available in BSL3 spaces (240). One screen in *M. marinum* utilized the antimicrobial effects of benzothiazoles and found both a novel synergistic drug as well as a potential protein target MMAR_0407 (Rv0164) (241). Another focused on targeting mycobacterial two-component signal transduction systems by combining computer-aided drug design with the *M. marinum* model. Other studies have focused on repurposing or enhancing existing drugs, such as antibiotic adjuvants (242–244). For example, a 2024 study utilized the *M. marinum-*zebrafish infection model to study bacterial survival and host cell response of a repurposed anti-cancer drug (242). Based on the strengths of the *M. marinum* model, it is well poised to continue contributing to these important fields.

## CONCLUSIONS

*M. marinum* has provided critical insight into mycobacterial pathogenesis, specifically between mycobacterium and the host. The development of molecular genetics in *M. marinum* has resulted in the identification of conserved virulence pathways and host responses essential for mycobacterial infection. The lower biosafety level and development of genetic tools in the *M. marinum* model organism have allowed large-scale genetic and proteomic screening for a deeper understanding of the regulation, virulence, and interaction with the host. In addition, the development of the zebrafish-*M. marinum* model for *M. tuberculosis* pathogenesis has significantly advanced the field through our understanding of the host-pathogen interactions in necrotic granulomas during complex, longer-term infections.
